# RPM-1 is localized to distinct subcellular compartments and regulates axon length in GABAergic motor neurons

**DOI:** 10.1186/1749-8104-9-10

**Published:** 2014-05-10

**Authors:** Karla J Opperman, Brock Grill

**Affiliations:** 1 Department of Neuroscience, The Scripps Research Institute - Florida, 130 Scripps Way, Jupiter, FL 33458, USA

**Keywords:** Axon termination, Neuronal development, RPM-1, PHR protein, Presynaptic terminal, SYD-2, Synapse formation, Synaptogenesis

## Abstract

**Background:**

The PAM/Highwire/RPM-1 (PHR) proteins are conserved signaling proteins that regulate axon length and synapse formation during development. Loss of function in *Caenorhabditis elegans rpm-1* results in axon termination and synapse formation defects in the mechanosensory neurons. An explanation for why these two phenotypes are observed in a single neuronal cell has remained absent. Further, it is uncertain whether the axon termination phenotypes observed in the mechanosensory neurons of *rpm-1* mutants are unique to this specific type of neuron, or more widespread defects that occur with loss of function in *rpm-1*.

**Results:**

Here, we show that RPM-1 is localized to both the mature axon tip and the presynaptic terminals of individual motor neurons and individual mechanosensory neurons. Genetic analysis indicated that GABAergic motor neurons, like the mechanosensory neurons, have both synapse formation and axon termination defects in *rpm-1* mutants. RPM-1 functions in parallel with the active zone component SYD-2 (Liprin) to regulate not only synapse formation, but also axon termination in motor neurons. Our analysis of *rpm-1*−/−; *syd-2*−/− double mutants also revealed a role for RPM-1 in axon extension. The MAP3K DLK-1 partly mediated RPM-1 function in both axon termination and axon extension, and the relative role of DLK-1 was dictated by the anatomical location of the neuron in question.

**Conclusions:**

Our findings show that axon termination defects are a core phenotype caused by loss of function in *rpm-1*, and not unique to the mechanosensory neurons. We show in motor neurons and in mechanosensory neurons that RPM-1 is localized to multiple, distinct subcellular compartments in a single cell. Thus, RPM-1 might be differentially regulated or RPM-1 might differentially control signals in distinct subcellular compartments to regulate multiple developmental outcomes in a single neuron. Our findings provide further support for the previously proposed model that PHR proteins function to coordinate axon outgrowth and termination with synapse formation.

## Background

During development, an axon uses long-range extracellular guidance cues to navigate the developmental landscape. Upon reaching its target site, the axon interprets guidepost signals from surrounding cells, and forms a chemical synapse [1,2]. Ultimately, the axon must also terminate outgrowth, which is a process referred to as axon termination, at the appropriate time and location.

The PAM/Highwire/RPM-1 (PHR) proteins play an important role during development, where they regulate axon length (dictated by a balance between axon extension and axon termination) and synapse formation [3]. In mice, Phr1 regulates synapse formation and axon extension in motor neurons [4,5], and regulates axon termination in sensory neurons [5]. In fish and mice, Phr1 also regulates axon guidance in the central nervous system [6-9].

Invertebrate systems have also informed our understanding of the PHR proteins. *Drosophila* Highwire (Hiw) regulates axon branching, and synapse formation at the neuromuscular junction [10,11]. The extensive overgrowth of motor axons in Hiw mutants suggests that axon termination is likely to be defective in these animals. Work using fly sensory neurons has also shown that Hiw regulates axon termination [12]. In *Caenorhabditis elegans*, the regulator of presynaptic morphology 1 (RPM-1) regulates synapse formation in motor neurons [13], and both axon termination and synapse formation in the mechanosensory neurons [14]. Studies in worms and flies have found that Hiw and RPM-1 function in axon guidance [15,16].

Because of anatomical differences between the motor axons of flies and worms (where termination sites are not easily observed, as a result of tiling), it has remained unclear whether RPM-1 regulates axon termination in motor neurons. This uncertainty has left open the question of whether axon termination defects are a widespread consequence of losing RPM-1 function, or a cell-specific phenotype associated with the mechanosensory neurons. Further, cell biological evidence has been lacking to help explain the diverse functional roles that the PHR proteins play during development.

Here, we show that RPM-1 regulates axon termination in the GABAergic motor neurons. Defective axon termination in the motor neurons of *rpm-1* loss of function (lf) mutants occurs in addition to defects in synapse formation. Of note, in some anatomical locations RPM-1 regulates both axon termination and axon extension of a single process. Transgenic analysis indicated that *rpm-1* functions cell autonomously to regulate axon termination, similar to synapse formation. This is consistent with our observation that RPM-1 localizes to both presynaptic terminals and the mature axon tip of individual motor neurons. Importantly, this is not an isolated subcellular distribution, as RPM-1 is also concentrated in the axon tip and presynaptic terminals of mechanosensory neurons. Thus, the subcellular location of RPM-1 is consistent with the presence of axon termination and synapse formation defects in both the motor neurons and the mechanosensory neurons of *rpm-1* (lf) mutants.

## Results

### *rpm-1* and *syd-2*/liprin function in parallel genetic pathways to regulate synapse formation in GABAergic motor neurons

*C. elegans* moves using sinusoidal body undulations. While an oversimplification [17], movement is generated by cholinergic activation of muscles on one side of the animal via the VA, DA, VB and DB neurons, and GABAergic inhibition of muscles on the opposing side via the ventral and dorsal D neurons (VDs and DDs) [18].

**Figure 1:**
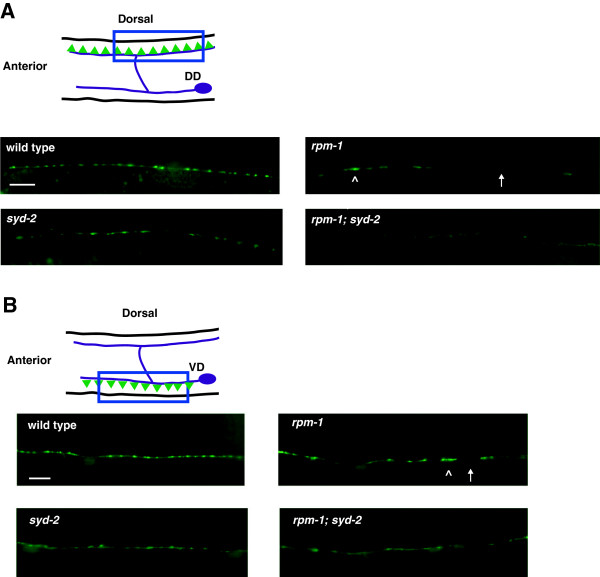
***rpm-1 *and *syd-2 *regulate synapse formation in GABAergic motor neurons. (A)** Schematic of a DD motor neuron innervating dorsal muscle cells (inspired by Worm Atlas). Green triangles represent presynaptic terminals. P_*unc-25*_SNB-1::GFP (*juIs1*) was visualized with epifluorescent microscopy for the indicated genotypes. The dorsal cord has gaps (arrow) and aggregated presynaptic terminals (arrowhead) in *rpm-1* mutants. **(B)** Schematic of VD motor neuron innervating ventral muscle cells. Green triangles represent presynaptic terminals. SNB-1::GFP was visualized with epifluorescent microscopy for the indicated genotypes. The ventral cord has gaps (arrow) and aggregated presynaptic terminals (arrowhead) in *rpm-1* mutants. In A and B, defects are enhanced in *rpm-1*; *syd-2* double mutants. Analysis was performed on young adults grown at 25°C. DD, dorsal D neuron; VD, ventral D neuron. Scale bar, 10 μm.

Each individual DD motor neuron (DD1 to DD6) extends a single axon that bifurcates. In adults, the ventral process receives neurotransmitter input, and a second process crosses the animal’s mid-body and forms presynaptic connections with the dorsal muscle cells (Figure 1A). The axons of the six DD neurons and their presynaptic sites are tiled contiguously along the dorsal cord [19]. The 13 VD neurons are arranged with an opposing orientation, and tile their presynaptic sites along the ventral cord (Figure 1B) [20]. A transgene, *juIs1*, which uses a cell-specific promoter to drive expression of a fusion protein of synaptobrevin-1 (SNB-1) and green fluorescent protein (GFP) (SNB-1::GFP) [21], was used to visualize the presynaptic terminals of the D neurons. In wild-type animals, SNB-1::GFP puncta were evenly distributed along the dorsal and ventral cords (Figure 1A,B). Consistent with previous observations [13], SNB-1::GFP puncta were aggregated and sections of the dorsal and ventral cords lacked puncta in *rpm-1−*/− mutants (Figure 1A,B). Importantly, electron microscopy has established that defects in SNB-1::GFP puncta in *rpm-1*−/− animals reflect defects in synapse formation, rather than simply defects in SNB-1 trafficking [13].

SYD-2 regulates active zone size, and defects in the active zone of *syd-2−*/− mutants result in abnormal morphology of SNB-1::GFP puncta in D neurons [22,23]. We also observed abnormal, diffuse morphology of SNB-1::GFP puncta in *syd-2−/− a*nimals in the dorsal (Figure 1A) and ventral cord (Figure 1B). Consistent with a previous study [24], we found that *rpm-1−/−*; *syd-2−/− *double mutants were uncoordinated and small (data not shown), and had enhanced defects in synapse formation in the dorsal (Figure 1A) and ventral cord (Figure 1B). Given that we used null alleles [13,22], these results confirm that *rpm-1* and *syd-2* function in parallel genetic pathways to regulate synapse formation in the GABAergic motor neurons.

### *rpm-1* and *syd-2* regulate axon termination and axon extension at the posterior tip of the dorsal cord

Aside from its role in synapse formation, RPM-1 also functions in the mechanosensory neurons to regulate axon termination [14,25]. Because the processes of the GABAergic motor neurons in *C. elegans* are tiled, termination points are not easily observed. As a result, it is uncertain whether *rpm-1* regulates axon termination in these neurons.

**Figure 2:**
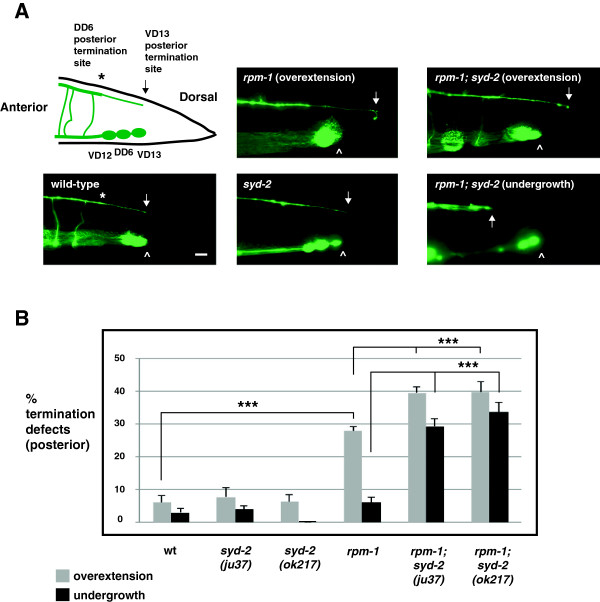
***rpm-1 *and *syd-2 *regulate posterior dorsal cord termination. (A)** Schematic of the posterior GABAergic motor neurons (VD12, DD6, and VD13) (inspired by Worm Atlas). The posterior tip of the dorsal cord was visualized using P_*unc-25*_GFP (*juIs76*) and epifluorescent microscopy. Highlighted are the posterior termination sites of DD6 (asterisk) and VD13 (arrow). The VD13 process terminates extension above the VD13 cell body (arrowhead). In *rpm-1* mutants, posterior termination is impaired and the dorsal cord is overextended. In *rpm-1*; *syd-2* double mutants, failed extension (undergrowth) and termination defects (overextension) were observed. Scale bar, 10 μm. **(B)** Quantitation of posterior termination defects for the indicated genotypes. For each genotype, the mean is shown from five or more counts (at least 20 worms/count). Analysis was performed on young adults grown at 23°C. Error bars represent the standard error of the mean. Significance was determined using an unpaired Student’s *t* test: ***P < 0.001.

Previous electron and light microscopy studies showed that the processes of DD6 and VD13 are fasciculated, and that the VD13 process extends alone to a stereotyped termination point at the posterior tip of the dorsal cord [19,26]. These observations suggested that if RPM-1 regulated axon termination in the DD or VD motor neurons, defects might be detected at the posterior tip of the dorsal cord. A transgene, *juIs76*, which uses a cell-specific promoter to drive expression of GFP, was used to visualize the morphology of the DD and VD neurons [27]. In wild-type *juIs76* animals, GFP is present throughout the nerve cords. We observed relatively precise termination of the dorsal cord at the posterior of the animal as a single, thin VD13 process, which was consistent with prior work (Figure 2A, arrow) [26]. The VD13 termination site corresponded consistently to the relative position of the VD13 cell body (Figure 2A, arrowhead). Anterior to this termination point, we observed a thicker bundle that reflects the DD6 termination point overlapping with the VD13 process (Figure 2A, asterisk). In *rpm-1−*/− mutants, we observed termination defects in which the posterior tip of the dorsal cord overextended beyond the position of the VD13 cell body (Figure 2A). Quantitation of overextension defects showed they were modestly penetrant, but significant (compare 27.9 ± 1.3% termination defects for *rpm-1* with 6.0 ± 2.2% for wild-type, Figure 2B). Given location and process thickness, these defects are likely to reflect overextension of the VD13 process.

Because *syd-2* functions in a parallel pathway with *rpm-1* to regulate synapse formation, we also tested whether *syd-2* regulates dorsal cord termination. Our analysis relied upon two alleles of *syd-2*: *ju37*, which results in a premature stop at glutamine 397 and is likely to be a molecular null allele [22], and *ok217*, which results in a stop codon at position 200 and, as assessed by RT-PCR and immunoblotting is likely to represent a null allele [28]. While *syd-2−*/− mutants did not have defects in posterior termination, *rpm-1−*/−; *syd-2−*/− double mutants had enhanced penetrance of overextension defects compared with *rpm-1−*/− single mutants (compare 39.6 ± 1.9% overextension termination defects for *rpm-1*; *syd-2*(*ju37*) and 39.8 ± 3.1% for *rpm-1*; *syd-2*(*ok217*) with 27.9 ± 1.3% for *rpm-1*, Figure 2A,B). Interestingly, *rpm-1−*/−; *syd-2−*/− double mutants also had posterior axon extension defects in which the dorsal cord was undergrown (Figure 2A). Axon undergrowth occurred with moderate, but significant, penetrance in *rpm-1−*/−; *syd-2−*/− double mutants (29.3 ± 2.4% undergrowth for *rpm-1*; *syd-2*(*ju37*) and 33.6 ± 3.0% for *rpm-1*; *syd-2*(*ok217*), Figure 2B). Thus, *rpm-1* regulates both axon termination and axon extension at the posterior tip of the dorsal cord by functioning in a parallel genetic pathway to *syd-2*. Our observation that axon extension phenotypes were only observed in *rpm-1−*/−; *syd-2−*/− double mutants and that axon termination phenotypes were present in *rpm-1−*/− single mutants supports two conclusions. (1) RPM-1 functions primarily in axon termination and secondarily in axon extension. (2) RPM-1 potentially regulates the balance between axon extension and axon termination in an individual neuron.

### *rpm-1* and *syd-2* regulate axon termination at the anterior tip of the dorsal cord

**Figure 3:**
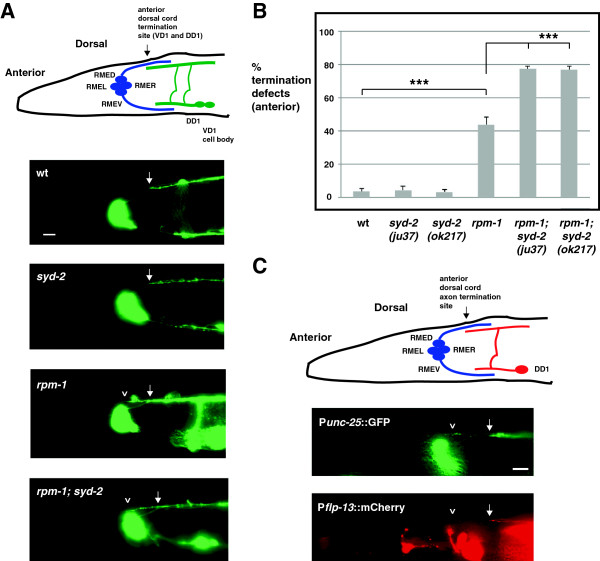
***rpm-1 *and *syd-2 *regulate anterior dorsal cord termination. (A)** Shown is a schematic of the DD1 and VD1 neurons that fasciculate to form the anterior tip of the dorsal cord, which terminates prior to the RMED axon (arrow) (inspired by Worm Atlas). The anterior tip of the dorsal cord was visualized using P_*unc-25*_GFP (*juIs76*) and epifluorescent microscopy. Highlighted are the normal termination site (arrow), and anterior overextension defects in *rpm-1* mutants and *rpm-1*; *syd-2* double mutants (arrowhead). **(B)** Quantitation of anterior termination defects for the indicated genotypes. For each genotype, the mean is shown from five or more counts (at least 20 worms/count). **(C)** P_*unc-25*_GFP (*juIs76*) and P_*flp-13*_mCherry (*bggIs6*) were visualized in *rpm-1* mutants using epifluorescent microscopy. Shown is a GFP positive, overextended dorsal cord process (upper panel, arrowhead), and the mCherry positive, anterior termination site of DD1 in a normal location (lower panel, arrow). Analysis was performed on young adults grown at 23°C. Error bars represent the standard error of the mean. Significance was determined using an unpaired Student’s *t* test: ***P < 0.001. RMED, dorsal RME neuron; RMEL, left RME neuron; RMER, right RME neuron; RMEV, ventral RME neuron; wt, wild-type. Scale bars, 10 μm.

Axon termination can also be assessed relatively easily at the anterior tip of the dorsal cord, which consists of the fasciculated processes of the DD1 and VD1 neurons. In wild-type animals, anterior termination occurs prior to the axon of the dorsal RME neuron (RMED) (Figure 3A, schematic and arrow). *rpm-1−*/− mutants showed termination defects in which the dorsal cord overextended beyond the RMED axon (Figure 3A, arrowhead). Overextension defects occurred with moderate penetrance in *rpm-1*−/− mutants (43.8 ± 4.4% termination defects, Figure 3B). In *syd-2*−/− animals, the anterior tip of the dorsal cord terminated extension normally (Figure 3A,B). However, the penetrance of anterior termination defects was enhanced in *rpm-1*−/−; *syd-2*−/− double mutants (compare 77.3 ± 1.7% termination defects for *rpm-1*; *syd-2*(*ju37*) and 76.9 ± 2.2 for *rpm-1*; *syd-2*(*ok217*) with 43.8 ± 4.4% for *rpm-1*, Figure 3B).

Anterior termination defects potentially reflected overextension of the processes of DD1 or VD1. To differentiate these two neurons, we simultaneously expressed two transgenes: *juIs76* (P_*unc-25*_GFP, expressed in the VD and DD neurons) and *bggIs6* (P_flp-13_mCherry, expressed only in the DD neurons [29]). As shown in Figure 3C, the anteriorly overextended process in *rpm-1−*/− mutants was labeled with GFP (*juIs76*), but not mCherry (*bggIs6*), demonstrating that this phenotype was probably due to overextension of the VD1 process.

### *rpm-1* and *syd-2* function cell autonomously to regulate dorsal cord termination and extension

**Figure 4:**
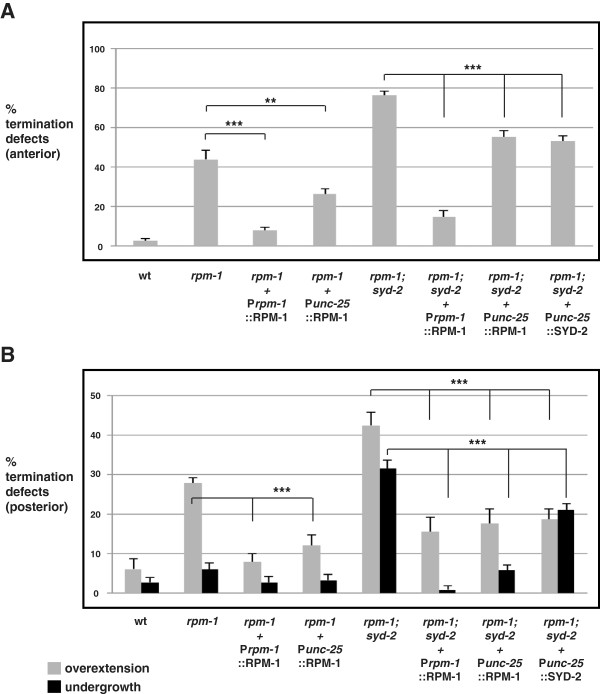
***rpm-1 *and *syd-2 *function cell autonomously to regulate anterior and posterior dorsal cord termination.** Quantitation of defects at **(A)** the anterior and **(B)** the posterior tip of the dorsal cord using P_*unc-25*_GFP (*juIs76*) for the indicated genotypes. For transgenes, the mean is shown for data collected from four or more independently derived transgenic lines for each genotype. Analysis was performed on young adults grown at 23°C. Error bars represent the standard error of the mean. Significance was determined using an unpaired Student’s *t* test: ***P <0.001, **P < 0.01. wt, wild-type.

*rpm-1* functions cell autonomously in the GABAergic motor neurons to regulate synapse formation [13] and in the mechanosensory neurons to regulate axon termination and synapse formation [14]. Similarly, *syd-2* functions cell autonomously in the GABAergic motor neurons to regulate active zone assembly [22]. To test whether *rpm-1* and *syd-2* function cell autonomously to regulate dorsal cord termination, we engineered *rpm-1−*/− single mutants or *rpm-1*−/−; *syd-2*−/− double mutants that carried a transgenic extrachromosomal array in which the native *rpm-1* promoter or a cell-specific promoter (P_*unc-25*_) was used to express RPM-1 or SYD-2. We found that transgenic expression of RPM-1 using its native promoter (P_rpm-1_RPM-1), which is only expressed in neurons, strongly rescued the anterior and posterior overextension defects in *rpm-1*−/− mutants and rescued the enhanced defects in *rpm-1*−/−; *syd-2*−/− double mutants (Figure 4A,B). Transgenic expression of RPM-1 specifically in the GABAergic motor neurons (P_*unc-25*_RPM-1) partially, but significantly, rescued the termination defects in *rpm-1*−/− mutants and *rpm-1*−/−; *syd-2*−/− double mutants (Figure 4A,B). Transgenic expression of SYD-2 specifically in the GABAergic motor neurons partially, but significantly, rescued anterior and posterior termination defects (overextension), and posterior extension defects (undergrowth) in *rpm-1*−/−; *syd-2*−/− double mutants (Figure 4A,B).

It should be noted that we tested a range of injection concentrations from 1 ng/μl to 20 ng/μl for transgenes driven by the *unc-25* promoter. For the posterior termination site, optimal results were obtained with DNA injected at 1 to 2.5 ng/μl. For the anterior termination site, optimal results were obtained with DNA injected at 5 ng/μl. Given the wide range of concentrations that we tested, the reduced efficacy of rescue with the *unc-25* promoter compared with the *rpm-1* promoter might reflect differences in timing of expression during development.

Overall, these findings demonstrate that lesions in *syd-2* and *rpm-1* are responsible for dorsal cord termination defects, and that *syd-2* and *rpm-1* function cell autonomously in the GABAergic motor neurons to regulate axon termination and axon extension at the anterior and posterior tip of the dorsal cord.

### RPM-1 functions through DLK-1, FSN-1 and GLO-4 to regulate dorsal cord termination and extension

Previous studies have identified several mechanisms by which RPM-1 regulates synapse formation and axon termination. (1) RPM-1 negatively regulates a mitogen activated protein (MAP) kinase pathway by ubiquitinating the most upstream kinase in the pathway, the dual leucine zipper-bearing kinase 1 (DLK-1) [30,31]. (2) RPM-1 functions as part of an E3 ubiquitin ligase complex that includes F-box synaptic protein 1 (FSN-1) [24]. (3) RPM-1 positively regulates gut granule loss 4 (GLO-4) and activates a Rab GTPase pathway [25]. (4) RPM-1 positively regulates the microtubule binding protein RAE-1 [32]. We wanted to test whether RPM-1 functions through similar mechanisms to control dorsal cord termination.

**Figure 5:**
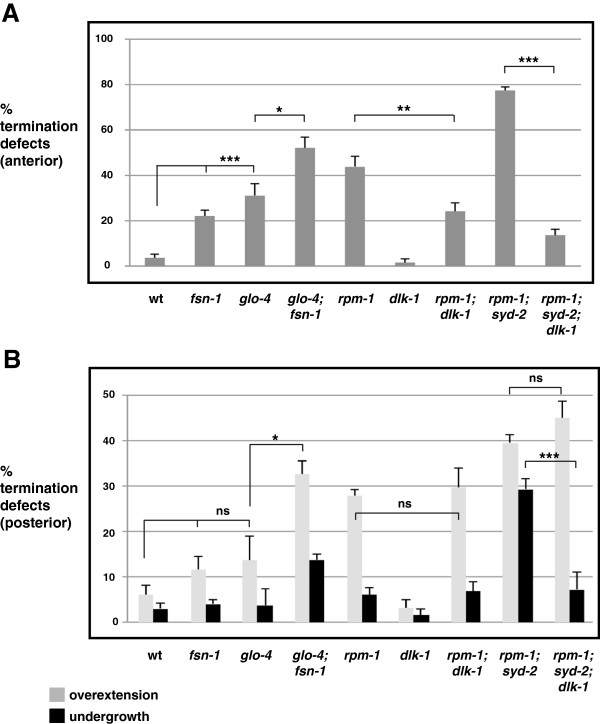
**Anterior and posterior dorsal cord termination is regulated by *fsn-1*, *glo-4*, and *dlk-1*.** Quantitation of termination defects at **(A)** the anterior and **(B)** the posterior tip of the dorsal cord using P_*unc-25*_GFP (*juIs76*) for the indicated genotypes. For each genotype, the mean is shown from five or more counts (at least 20 worms/count). Analysis was performed on young adults grown at 23°C. Error bars represent the standard error of the mean. Significance was determined using an unpaired Student’s *t* test: ***P <0.001 **P < 0.005, *P < 0.05, ns, not significant. wt, wild-type.

In *glo-4*−/− and *fsn-1*−/− single mutants, anterior termination defects were present, but they occurred with lower penetrance than in *rpm-1*−/− mutants (Figure 5A). In contrast, *glo-4*−/− and *fsn-1*−/− single mutants did not display significant posterior termination defects (Figure 5B). We observed enhanced penetrance of anterior and posterior termination defects in *glo-4*−/−; *fsn*−/− double mutants that were of similar levels to *rpm-1*−/− mutants (Figure 5A,B). Because we used null alleles of *glo-4* and *fsn-1*, these results show that *glo-4* and *fsn-1* function in parallel genetic pathways to regulate termination of the dorsal cord. Prior studies have shown that FSN-1 and GLO-4 mediate RPM-1 function in axon termination in the mechanosensory neurons and synapse formation in the GABAergic motor neurons [24,25]. Our results support the model that RPM-1 functions through similar mechanisms to regulate axon termination in GABAergic motor neurons.

Next, we examined the MAP kinase kinase kinase (MAP3K) DLK-1, which also functions downstream of RPM-1 and is a target of the ubiquitin ligase activity of RPM-1 [25,30]. *dlk-1*−/− single mutants displayed normal anterior and posterior termination (Figure 5A,B). However, in *rpm-1*−/−; *dlk-1*−/− double mutants and in *rpm-1*−/−; *syd-2*−/−; *dlk-1*−/− triple mutants, we observed strong, but partial suppression of anterior termination defects (Figure 5A). In contrast, posterior overextension defects were not suppressed in *rpm-1*−/−; *dlk-1*−/− double mutants or *rpm-1*−/−; *syd-2*−/−; *dlk-1*−/− triple mutants (Figure 5B). Interestingly, *rpm-1*−/−; *syd-2*−/−; *dlk-1*−/− triple mutants showed strong suppression of undergrowth defects in the posterior tip of the dorsal cord (Figure 5B). Thus, in GABAergic motor neurons DLK-1 plays a varying role in mediating the function of RPM-1 in axon termination, and anatomical location appears relevant to the role of DLK-1. Importantly, our data also indicate that DLK-1 regulates axon termination in the anterior of the dorsal cord, and regulates extension (and not termination) in the posterior of the dorsal cord.

### *rpm-1* regulates axon termination of the DD5 neuron within the dorsal cord

**Figure 6:**
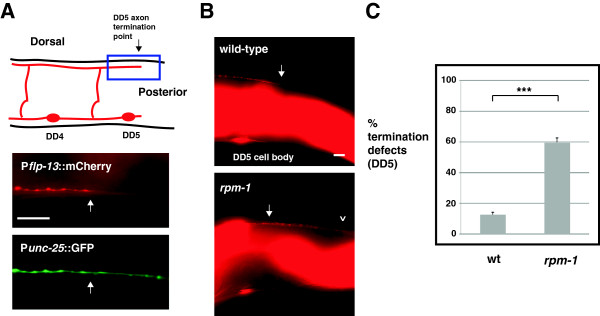
***rpm-1 *regulates axon termination of the DD5 motor neuron. (A)** Schematic highlights the axon termination site of the DD5 neuron (arrow) (inspired by Worm Atlas). Blue box highlights the region of the DD5 axon that was visualized using epifluorescent microscopy and two transgenes: P_*unc-25*_GFP (*juIs76*) and P_*flp-13*_mCherry (*bggIs6*). mCherry highlights the DD5 termination point (arrow), while GFP fills both DD5 and DD6. **(B)** Arrow highlights the normal DD5 axon termination point. In *rpm-1* mutants, the DD5 axon overextends (arrowhead). **(C)** Quantitation of DD5 axon termination defects for the indicated genotypes. For each genotype, the mean is shown from five or more counts (at least 20 worms/count). Analysis was performed on young adults grown at 23°C. Error bars represent the standard error of the mean. Significance was determined using an unpaired Student’s *t* test: ***P < 0.001. Scale bars, 10 μm. DD, dorsal D neuron; wt, wild-type.

Given that *rpm-1* regulates axon termination at the tips of the dorsal cord, we next sought to determine whether *rpm-1* regulates axon termination within the interior of the dorsal cord. To address this question, we used the transgene *bggIs6* (P_flp-13_mCherry) that expresses mCherry only in the DD neurons, which have axons that tile along the length of the dorsal cord and innervate dorsal muscles in adults [20]. In wild-type *bggIs6* animals, mCherry was clearly visible in the DD neurons from DD1 (most anterior DD neuron) to DD5 (interior DD neuron). mCherry expression in DD6 (most posterior DD neuron) was insufficient for detection by epifluorescent microscopy. We confirmed that mCherry was expressed in DD5 and not detected in DD6 by analyzing animals that were transgenic for both *juIs76* (labeled all DD and VD neurons with GFP) and *bggIs6* (labeled only DD1 to 5 with mCherry) (Figure 6A).

In wild-type animals, the DD5 axon consistently terminated at a location that corresponded to the ventral termination site of the small, posterior DD5 process (Figure 6A schematic and 6B, arrow). In contrast, *rpm-1*−/− mutants had axon termination defects in which the DD5 axon overextended beyond its normal termination point (Figure 6B, arrowhead). Axon termination defects in DD5 were moderately penetrant in *rpm-1−/− *animals (compare 59.7 ± 2.9% termination defects in *rpm-1* with 12.4 ± 1.8% in wild-type, Figure 6C). Thus, *rpm-1* regulates termination of the DD5 axon within the dorsal cord.

### RPM-1 localizes to the mature axon tips and the presynaptic terminals of GABAergic motor neurons and mechanosensory neurons

In motor neurons, RPM-1 localizes to the perisynaptic zone, a presynaptic region that surrounds the synaptic vesicles [13,25,33]. In *Drosophila*, transgenically expressed Hiw localizes broadly throughout the presynaptic terminal [34]. Localization of RPM-1 and Hiw to the presynaptic terminal is consistent with their function in synapse formation. While the localization of mammalian Phr1 in mature axons is unclear, in immature growing axons that lack synapses, murine Phr1 is localized throughout the axon, and excluded from much of the growth cone [5]. This observation suggested that PHR proteins might terminate extension by localizing to axon tips. Consistent with this hypothesis, RPM-1 is localized to puncta throughout the axon of SAB motor neurons, and is also concentrated at the tip of the SAB axon [33].

**Figure 7:**
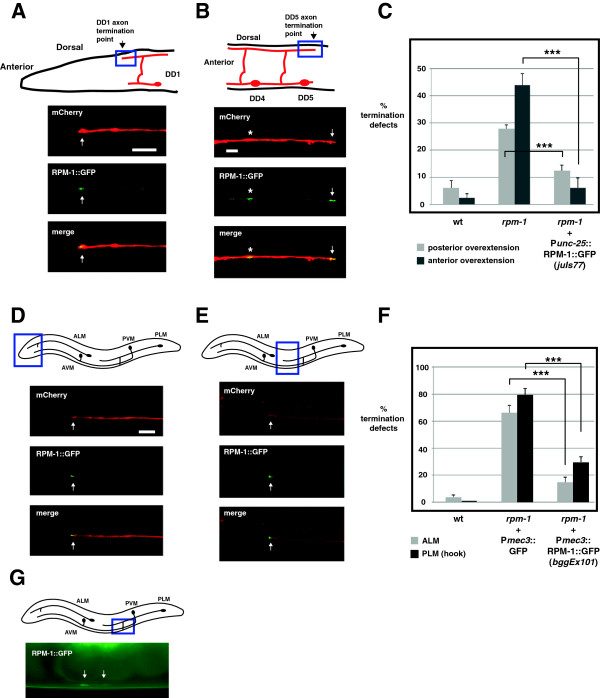
**RPM-1 localizes to the axon tips and presynaptic terminals of GABAergic motor neurons and mechanosensory neurons. (A)** Schematic shows the termination site of the DD1 neuron (arrow) (inspired by Worm Atlas). Blue box highlights where confocal microscopy was used to visualize P_*unc-25*_RPM-1::GFP (*juIs77*) and P_*flp-13*_mCherry (*bggEx99*). RPM-1::GFP is concentrated at the tip of the DD1 axon (arrow). **(B)** Schematic shows the morphology and termination site of the DD5 neuron. Blue box highlights where confocal microscopy was used to visualize P_*unc-25*_RPM-1::GFP (*juIs77*) and P_*flp-13*_mCherry (*bggEx99*). RPM-1::GFP is concentrated at presynaptic terminals (asterisk) and the tip of the DD5 axon (arrow). **(C)** P_*unc-25*_RPM-1::GFP (*juIs77*) rescues axon overextension defects caused by *rpm-1* (lf) in the posterior and anterior tip of the dorsal cord. For each genotype, the mean is shown from five or more counts (at least 20 worms/count). **(D**,**E)** Schematic shows the mechanosensory neurons of *C. elegans*. Blue box highlights the region of the animal where confocal microscopy was used to visualize P_*mec-7*_mCherry or P_*mec-3*_RPM-1::GFP (*bggEx101*). RPM-1::GFP is concentrated at the tip (arrow) of **(D)** the ALM axon and **(E)** the PLM axon. **(F)** P_*mec-3*_RPM-1::GFP (*bggEx101*) rescues axon termination defects in ALM (gray) and PLM neurons (hook, black bars). For each genotype, the mean is shown from three or more counts (at least 20 worms/count). For P_mec-3_GFP (negative control), three independently derived transgenic lines were analyzed. **(G)** Blue box highlights the region of the PLM that was visualized by epifluorescent microscopy. Shown below is RPM-1::GFP (*bggEx101*) concentrated at the presynaptic terminals of a PLM neuron (arrows). All images and analysis were generated using young adult animals grown at 23°C. Error bars represent the standard error of the mean. Significance was determined using an unpaired Student’s *t* test: ***P < 0.001. ALM, anterior lateral microtubule; AVM, anterior ventral microtubule; PLM, posterior lateral microtubule; PVM, posterior ventral microtubule; wt, wild-type. Scale bars, 5 μm.

Localization of RPM-1 to the perisynaptic zone of motor neurons is consistent with RPM-1 regulating synapse formation. However, this subcellular localization does not explain the role we have now discovered for RPM-1 in axon termination. To determine whether RPM-1 is localized to the axon tips of the DD neurons (GABAergic motor neurons) as well as to their presynaptic terminals, we used transgenics and confocal microscopy. *rpm-1*−/− animals were engineered to carry two transgenic arrays simultaneously: *juIs77* (an integrated array that expresses RPM-1::GFP in the VD and DD neurons), and *bggEx99* (an extrachromosomal array that expresses mCherry only in the DD neurons). In *juIs77*; *bggEx99* animals, mCherry allowed us to visualize the axon termination points of DD1 and DD5, where we readily observed concentration of RPM-1::GFP (Figure 7A,B, arrows). We also observed RPM-1::GFP puncta that were not at the axon termination point, and represent presynaptic localization (Figure 7B, asterisk). It should be noted that because RPM-1::GFP was expressed using the *unc-25* promoter, RPM-1::GFP puncta could be present in either the DD or VD neurons. However, the precise colocalization between RPM-1::GFP puncta, and DD1 and DD5 axon tips suggests that at least a portion of the RPM-1::GFP is likely to be localized at the DD axon tips. Importantly, *juIs77* rescued both axon termination and synapse formation defects in the DD motor neurons of *rpm-1*−/− mutants, thereby demonstrating that *juIs77* expresses a functional RPM-1::GFP construct that is expressed at physiologically relevant levels (Figure 7C and Additional file 1).

Previous studies showed that *rpm-1*−/− mutants have multiple defects in the mechanosensory neurons of *C. elegans*, including axon termination defects in the anterior lateral microtubule (ALM) and posterior lateral microtubule (PLM) neurons, and synaptic branch defects in the PLM neurons that are associated with a failure to form synapses [14,25]. While RPM-1 plays an important, cell autonomous function in the mechanosensory neurons, its subcellular localization in these neurons is unknown. Our observation that RPM-1 was concentrated at both axon termination sites and presynaptic terminals in the DD neurons suggested that this might also be the case in the mechanosensory neurons. To test this, we engineered *rpm-1*−/− animals that expressed a transgenic extrachromosomal array, *bggEx101,* that uses cell-specific promoters to express mCherry (P_mec-7_mCherry) and RPM-1::GFP (P_mec-3_RPM-1::GFP) simultaneously in the mechanosensory neurons. We observed that mCherry diffusely filled the axon, axonal branch, and cell bodies of the ALM and the PLM mechanosensory neurons (Figure 7D,E, and data not shown). In contrast, RPM-1::GFP was strongly concentrated in puncta at the terminal tip of both the ALM and PLM axons (Figure 7D,E, arrows). RPM-1::GFP was also concentrated at the presynaptic terminals of the PLM mechanosensory neurons (Figure 7G, arrows). We observed diffuse low levels of RPM-1::GFP in the ALM and PLM cell bodies, which was often excluded from the nucleus (data not shown). The intensity and location of our transgenic coinjection marker (P_ttx-3_RFP) prevented us from determining whether RPM-1::GFP was localized to the presynaptic terminals of the ALM neurons, but given the results in PLM neurons this is likely to be the case. *bggEx101* (P_mec-3_RPM-1::GFP) rescued both the ALM and the PLM axon termination defects in *rpm-1*−/− mutants, which demonstrates that this array expresses functional RPM-1::GFP at physiologically relevant levels (Figure 7F). Notably, while *bggEx101* was generated by injecting plasmid DNA encoding RPM-1::GFP at 20 ng/μl, arrays made with higher concentrations of DNA (50 ng/μl) often resulted in mechanosensory neurons with high levels of RPM-1::GFP expression. In such cells, GFP signal filled the entire cell body and axon, and concentration at the axon tip and at the presynaptic terminal could not be observed (data not shown).

These results, showing that RPM-1 is compartmentalized in discrete subcellular locations in the GABAergic motor neurons and the mechanosensory neurons, is consistent with the phenotypes caused by *rpm-1* (lf) in these types of neurons.

### Synaptic activity differentially regulates termination at the anterior and posterior tip of the dorsal cord

**Figure 8:**
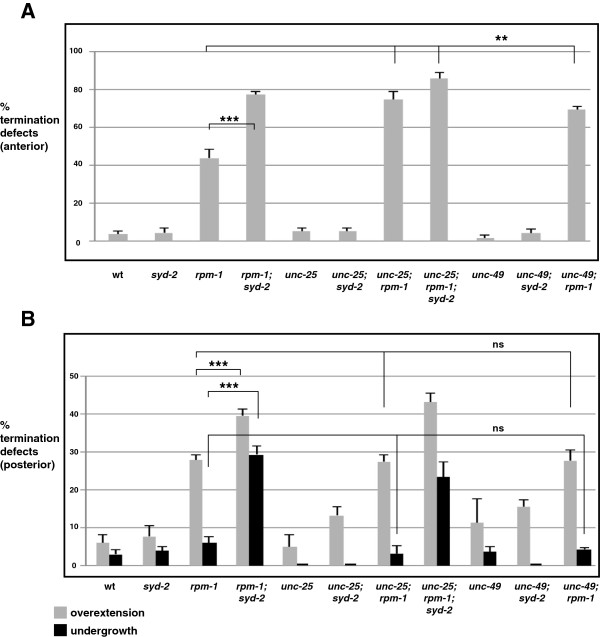
**Synaptic activity regulates termination at the anterior, but not the posterior, tip of the dorsal cord.** Quantitation of termination defects at **(A)** the anterior and **(B)** the posterior tip of the dorsal cord using P_*unc-25*_GFP (*juIs76*) for the indicated genotypes. For each genotype, the mean is shown from five or more counts (at least 20 worms/count). Analysis was performed on young adults grown at 23°C. Error bars represent the standard error of the mean. Significance was determined using an unpaired Student’s *t* test. ***P <0.001, **P < 0.005, ns, not significant. wt, wild-type.

Over 20 years ago, work on *Drosophila* indicated that axon length and branching in motor neurons was regulated by synaptic activity [35]. *Ether a-go-go*; *Shaker* double mutants show similar (although somewhat weaker) phenotypes to those observed in *Highwire* mutants, in which motor axons are overgrown and have excess branching, and abnormal synapse morphology is also observed. This suggested that the enhanced defects in dorsal cord termination observed in *rpm-1*−/−; *syd-2*−/− double mutants (Figures 2 and 3) might be due to loss of synaptic activity. This hypothesis was consistent with our observation that synapse formation was heavily impaired in the dorsal and ventral cords of *rpm-1*−/−; *syd-2*−/− double mutants (Figure 1). It is plausible that synaptic activity, synaptic connectivity, or a combination of both, might influence intracellular signals that regulate axon termination in the dorsal cord. We opted to test the role of synaptic activity in dorsal cord termination by impairing GABAergic transmission at the presynaptic terminal using *unc-25*−/− mutants, which lack glutamic acid decarboxylase [36], and by impairing transmission at the postsynaptic terminal using *unc-49*−/− mutants, which lack γ-amino butyric acid (GABA) receptors in muscles [18,37]. In *unc-25*−/− and *unc-49*−/− single mutants, we observed no defects in the morphology of GABAergic motor neurons (data not shown), or termination of the anterior or posterior tip of the dorsal cord (Figure 8A,B). Thus, loss of chemical neurotransmission at the GABAergic neuromuscular junction in *C. elegans* does not impair dorsal cord termination. Likewise, *unc-25*−/−; *syd-2*−/− and *unc-49*−/−; *syd-2*−/− double mutants did not have impaired anterior or posterior termination (Figure 8A,B). However, *unc-25*−/−; *rpm-1*−/− and *unc-49*−/−; *rpm-1*−/− double mutants showed enhanced penetrance of anterior termination defects similar to *rpm-1*−/−; *syd-2*−/− double mutants (Figure 8A). No further increase in defective anterior termination was observed in *unc-25*−/−; *rpm-1*−/−; *syd-2*−/− triple mutants (Figure 8A). In contrast, *unc-25*−/−; *rpm-1*−/− or *unc-49*−/−; *rpm-1*−/− double mutants did not enhance posterior termination defects, although *rpm-1*−/−; *syd-2*−/− double mutants were strongly enhanced for both overextension and undergrowth defects (Figure 8B). Thus, *rpm-1* functions coordinately with synaptic activity to regulate termination of the anterior, but not the posterior, tip of the dorsal cord.

## Discussion

The PHR proteins function in a range of developmental events, including axon termination, axon guidance, and synapse formation. In *C. elegans*, RPM-1 functions cell autonomously to regulate synapse formation in the GABAergic motor neurons, and functions cell autonomously in the mechanosensory neurons to regulate both axon termination and synapse formation [13,14,25,30]. We now show that RPM-1 also functions cell autonomously in the GABAergic motor neurons to regulate axon termination. Prior to our study, axon termination defects caused by *rpm-1* (lf) might have been considered cell-specific defects associated with the mechanosensory neurons and, as such, of lesser interest. Our results here demonstrate that this is not the case, and strengthen the argument that a core function of the PHR proteins is to regulate axon termination.

Our observation that RPM-1 is concentrated in two distinct subcellular compartments, the mature axon tip and the presynaptic terminal, points to a possible explanation for why RPM-1 regulates both axon termination and synapse formation in an individual neuron. RPM-1 may differentially regulate specific local signals or the intensity of core signals at different subcellular locations. This idea is consistent with a prior study, which showed that RPM-1 negatively regulates signaling by UNC5 (UNC-5) and Robo (SAX-3) to control axon termination in the mechanosensory neurons [16]. Thus, RPM-1 localized at the axon tip may regulate local signaling triggered by axon guidance cues. In contrast, RPM-1 signaling at the presynaptic terminal is unlikely to regulate signaling by guidance cues, and therefore presumably has a distinct role in synaptogenesis. It was previously proposed that RPM-1 might coordinate axon extension and termination with synapse formation [3,16]. Our finding that RPM-1 is localized to distinct subcellular compartments within individual neurons provides cell biological evidence to support this model further, and raises the interesting possibility that different activating or inhibitory signals might converge on RPM-1 in distinct locations.

Because RPM-1 is concentrated at the mature axon tip, it is possible that RPM-1 acts to cap a growing axon and trigger termination of extension. Presumably, localization of RPM-1 at the mature axon cap continues to silence signaling (most likely by guidance cues, as discussed previously) to ensure that the axon termination site is maintained. Transgenic studies in flies have shown that Hiw is enriched at presynaptic boutons, and there is a presynaptic bouton at the tip of fly motor neurons [34]. Thus, it is plausible that Hiw is concentrated at the mature axon tip, although this has not been explicitly examined. To our knowledge, it remains uncertain whether vertebrate PHR proteins are concentrated at the mature axon tip; however, work in mice has shown that Phr1 is localized throughout the axon and excluded from portions of the growth cone in actively growing axons that have not formed synapses [5]. Collectively, these observations raise the interesting possibility that at some point during development, PHR proteins may concentrate in the growth cone to trigger formation of a mature axon cap that is no longer capable of extension. Further support for or against this model is likely to be obtained by addressing a number of questions. Does RPM-1 localize to the axon tip prior to or following termination of axon outgrowth? What is the temporal relationship between RPM-1 localized to the axon tip and RPM-1 localized to the presynaptic terminal? Finally, where does RPM-1 localize during active axon growth prior to termination?

### *rpm-1* regulates axon termination and axon extension

Our genetic analysis showed that *rpm-1* (lf) mutants have defects in axon termination of the GABAergic motor neurons at the anterior tip, the posterior tip, and within the dorsal cord. *rpm-1*−/−; *syd-2*−/− double mutants had enhanced axon termination defects (evident by increased numbers of axons showing overgrowth), and also had enhanced defects in axon extension exclusively at the posterior tip of the dorsal cord (evident by increased numbers of neurons with axon undergrowth). Thus, the use of a sensitizing genetic background has allowed us to determine that *rpm-1* functions primarily in axon termination and secondarily in axon extension in the motor neuron (VD13) that forms the posterior dorsal cord termination site. To our knowledge, this is the first evidence that, in a single cell*, rpm-1* regulates both axon termination and extension of the same process. This provides further support for the model that RPM-1 is a general and key regulator of axon length.

Previous studies with fish cortical neurons and with murine motor neurons reported the surprising and differing result that Phr1 regulates microtubule disassembly and assembly, respectively [5,38]. Initially, we assumed that this paradox was due to a difference in the type of neuron analyzed. While this is still a potential factor, our finding that *rpm-1* regulates axon termination at the anterior and posterior tip of the dorsal cord, but regulates extension exclusively at the posterior, suggests the interesting possibility that the location of a neuron and its environment might also instruct how the PHR proteins regulate axon length. However, given the anatomical differences between different GABAergic motor neurons (for example, the VD1 neuron has unique axon anatomy), it remains possible that intrinsic differences in individual motor neurons dictate whether RPM-1 regulates axon extension or termination.

We have also found that synaptic activity, acting as a secondary player, functions coordinately with RPM-1 to regulate termination of the anterior tip of the dorsal cord. Thus, enhanced termination defects at the anterior of the dorsal cord in *rpm-1*−/−; *syd-2*−/− double mutants are likely to reflect enhancer effects associated with loss of synaptic transmission resulting from severely impaired synapse formation in these double mutants. By contrast, synaptic activity does not function coordinately with RPM-1 to regulate termination at the posterior tip of the dorsal cord. As a result, the enhanced defects in posterior termination of *rpm-1*−/−; *syd-2*−/− double mutants are likely to reflect loss of a signal other than synaptic transmission, possibly synaptic connectivity. Given that SYD-2 is localized to the active zone of presynaptic terminals and not axon tips [22,33], it is unlikely that SYD-2 functions at the axon tip to regulate axon termination. Overall, our results demonstrate that axon termination is established coordinately by a core signal from RPM-1, and secondary signals (such as synaptic activity), which are dependent upon the location or the type of neuron in question.

Prior studies showed that two Wnts, LIN-44 and EGL-20, and the canonical β-catenin BAR-1 regulate axon termination of the GABAergic motor neurons at the posterior tip of the dorsal cord [26] and within the dorsal cord [39]. Wnts acting in the posterior of *C. elegans* have also been shown to regulate synapse position in the cholinergic DA9 motor neuron [40], and axon polarization in the PLM mechanosensory neurons [41-43]. Given that both Wnt (lf) mutants, and *rpm-1* (lf) mutants have axon termination defects at the posterior tip of the dorsal cord, it is plausible that Wnt and RPM-1 signaling function together to regulate axon termination in GABAergic motor neurons. Consistent with this, we have found that *bar-1* functions in the same genetic pathway as *rpm-1* to regulate axon termination in the PLM mechanosensory neurons, and synapse formation in the GABAergic motor neurons (Tulgren and Grill, unpublished observation). In the future, we hope to address the question of whether RPM-1 and Wnt signaling converge differentially on BAR-1, thereby providing multiple mechanisms for regulation of the BAR-1 β-catenin.

### The role of DLK-1 in axon termination and axon extension varies with location

Previous studies established the role of RPM-1, and PHR proteins in general, as negative regulators of the MAP3K DLK-1 (called Wallenda in flies and Dlk in mammals) [5,11,30,44]. We have found that axon termination defects caused by *rpm-1* (lf) are suppressed by *dlk-1* (lf) at the anterior, but not the posterior tip of the dorsal cord. However, it is notable that *dlk-1* (lf), while unable to suppress enhanced overextension defects in *rpm-1*−/−; *syd-2*−/− double mutants, strongly suppressed undergrowth defects in *rpm-1*−/−; *syd-2*−/− double mutants. These results demonstrate that RPM-1 regulates axon extension at the posterior tip of the dorsal cord by inhibiting DLK-1, but RPM-1 does not function through DLK-1 to regulate axon termination in this location. Our findings are consistent with prior studies, which showed that RPM-1 and Hiw function only in part through DLK-1 signaling [11,25,34]. Further, our results suggest that the anatomical location of a neuron may dictate whether DLK-1 regulates axon termination or axon extension. We propose three possible explanations for the role that location plays in the variable contribution of DLK-1 to axon termination or extension. (1) Extracellular cues may differentially regulate RPM-1 effects on DLK-1. (2) The extracellular environment may shape the relative contribution of DLK-1 signaling or the activation of DLK-1 independent of RPM-1. (3) Intrinsic differences between GABAergic motor neurons in different anatomical locations may affect the contribution of DLK-1 signaling to axon termination and extension.

### *rpm-1* regulates both axon termination and synapse formation in GABAergic motor neurons

Our analysis indicated that the anterior and posterior dorsal cord termination defects in *rpm-1* (lf) mutants probably reflect overextension of the VD1 and VD13 processes, respectively. In the interior of the dorsal cord, *rpm-1* (lf) mutants have axon termination defects in the DD5 motor neuron. Previous work showed that synapse formation defects are also observed along the length of the dorsal cord in *rpm-1* (lf) mutants and, thus, are occurring in DD5 [13,30]. The presence of both axon termination defects and synapse formation defects in the DD5 neuron of *rpm-1* (lf) mutants is consistent with our observation that RPM-1 is localized to the axon tip and the presynaptic terminal of DD5 (Figure 7B). Thus, RPM-1 regulates both axon termination and synapse formation in a single motor neuron, DD5. Similar logic suggests that the same situation exists in VD1 and VD13.

## Conclusions

Our findings prompt several conclusions. (1) RPM-1 is localized to distinct subcellular compartments at mature axon tips and presynaptic terminals, which is consistent with RPM-1 regulating axon termination and synapse formation, respectively. (2) RPM-1 functions coordinately with different signals, one of which is synaptic activity, to regulate axon termination in different anatomical locations. (3) As the relative success of synapse formation is reduced (such as in *rpm-1*−/−; *syd-2*−/− double mutants compared with *rpm-1*−/− single mutants), the penetrance of axon termination defects increases, suggesting a molecular link between these two processes. Collectively, these findings raise an intriguing possibility: RPM-1 may function in different subcellular locations to coordinate synapse formation with termination of axon outgrowth, based on the level of synaptic activity or connectivity.

## Methods

### Strains and genetics

The N2 strain of *C. elegans* was propagated using standard techniques [45]. Alleles used included: *rpm-1*(*ju44*), *syd-2*(*ju37*), *syd-2*(*ok217*), *fsn-1*(*gk429*), *glo-4*(*ok623*), *dlk-1*(*ju476*), *unc-25*(*e156*), and *unc-49*(*e362*). Standard *C. elegans* genetic procedures were used to generate double and triple mutants, and alleles were tracked by PCR genotyping. Because PCR could not be used for *syd-2*(*ju37*), we used an X-linked mating strategy. *syd-2*; *rpm-1* double mutants were identified based on their UncSma (Uncoordinated and Small) phenotype. *syd-2*; *rpm-1*; *dlk-1* triple mutants were constructed by isolating *rpm-1*−/−; *syd-2*−/−; *dlk-1*+/−animals that were UncSma, and subsequently isolating homozygous triple mutants in which the UncSma phenotype was suppressed. *juIs77*/+*juIs76*/+recombinant heterozygous animals were isolated by visually monitoring both *juIs76* and P_ttx-3_GFP expressed by *juIs77*. These animals were analyzed as heterozygotes because *juIs77*; *juIs76* homozygous animals were not viable, for reasons that were unclear. Transgenes used included: *juIs1* (P_*unc-25*_SNB-1::GFP), *juIs76* (P_*unc-25*_GFP), *juIs77* (P_*unc-25*_RPM-1::GFP), *bggEx99* (P_*flp-13*_mCherry), *bggIs6* (P_*flp-13*_mCherry), and *bggEx101* (P_*mec-*3_RPM-1::GFP; P_*mec-7*_mCherry). Work and protocols performed with *C. elegans* involving recombinant DNA were approved by the Institutional Biosafety Committee of The Scripps Research Institute - Florida (protocol #: 2012-013).

### Transgenics

Transgenic animals were generated by standard microinjection procedures. Transgenes were constructed using the coinjection marker P_ttx-3_RFP (50 ng/μl), and pBluescript (50 ng/μl). For transgenic rescue experiments, plasmids included: pCZ160 (P_rpm-1_RPM-1), pBG-46 (P_mec-3_RPM-1::GFP), pBG-137 (P_*unc-25*_RPM-1), pBG-GY465 (P_*unc-25*_SYD-2), and pBG-GY497 (P_mec-3_GFP). pCZ160 (P_rpm-1_RPM-1), pBG-46 (P_mec-3_RPM-1::GFP), and pBG-137 (P_*unc-25*_RPM-1) were injected into *rpm-1*−/− mutants at 20 ng/μl for all rescues. pBG-GY465 (P_*unc-25*_SYD-2) was injected into *rpm-1*−/−; *syd-2*−/− double mutants at 5 ng/μl for posterior and anterior overextension defects, and at 1 to 2.5 ng/μl for posterior undergrowth defects. For *bggEx99*, pBG-GY411 (P_flp-13_mCherry) was injected at 50 ng/μl. *bggIs6* was a spontaneous integrant of *bggEx99*. For *bggEx101*, pBG-46 (P_mec-3_RPM-1::GFP) and pBG-GY258 (P_mec-7_mCherry) were injected at 20 ng/μl and 10 ng/μl, respectively.

### Analysis of synapse and axonal morphology using epifluorescent microscopy

For analysis of GFP, mCherry or SNB-1::GFP, live animals were anesthetized using 1% (v/v) 1-phenoxy-2-propanol in M9 buffer and visualized using a 40× magnification oil-immersion lens and an epifluorescent microscope (Leica CFR5000). A CCD camera (Leica DFC345 FX) was used for documentation. Images were analyzed and scale bars applied using Leica Application Suite for Advanced Fluorescence (LAS-AF) software. Termination defects were quantified by manually scoring *juIs76* (P_*unc-25*_::GFP) for dorsal cord tips, and *bggIs6* (P_*unc-25*_::mCherry) for DD5. Each genotype was quantitated by manually scoring three counts of 10 to 20 animals from two or more independent experiments.

### Confocal microscopy

Transgenic animals were anesthetized using 1% (v/v) 1-phenoxy-2-propanol in M9 buffer. GFP::RPM-1 and mCherry were visualized in live animals using a Zeiss 780 laser scanning confocal microscope at 63× magnification under oil immersion. Images were acquired using Zeiss’ ZEN software, and analyzed using Image J software.

## Abbreviations

ALM: anterior lateral microtubule; CCD: charge-coupled device; DD: dorsal D neuron; DLK: duel leucine zipper-bearing kinase; FSN: F-box synaptic protein; GABA: γ-amino butyric acid; GFP: green fluorescent protein; GLO: Gut granule loss; Hiw: Highwire; lf: loss of function; MAP3K: mitogen activated protein kinase kinase kinase; PAM: protein associated with Myc; PCR: polymerase chain reaction; PHR: PAM/Highwire/RPM-1; PLM: posterior lateral microtubule; RMED: dorsal RME neuron; Robo: roundabout; RPM: regulator of presynaptic morphology; RT-PCR: reverse transcriptase polymerase chain reaction; SAX: sensory axon guidance; SNB: synaptobrevin; SYD: synapse defective; UNC: uncoordinated; VD: ventral D neuron.

## Competing interests

The authors declare that they have no competing interests.

## Authors’ contributions

KJO performed experiments and generated genetic and transgenic *C. elegans* strains. BG designed and performed experiments, analyzed data, performed statistical analysis, and wrote the manuscript. Both authors read and approved the final manuscript.

## Supplementary Material

Additional file 1 ***juIs77* (P**_***unc-25***_**RPM-1::GFP) rescues synapse formation defects in *rpm-1* mutants.***juIs1* (P_*unc-25*_SNB-1::GFP) was used to visualize presynaptic terminals in the dorsal cords of animals with the indicated genotypes. SNB-1::GFP puncta were quantitated by scoring the number of puncta that were present per 100 μm of dorsal cord. Note that *juIs77* (P_*unc-25*_RPM-1::GFP rescues synapse formation defects caused by *rpm-1* (lf). Click here for file
