# Supplementary figures and images for: RPM-1 is localized to distinct subcellular compartments and regulates axon length in GABAergic motor neurons
Source: Neural Dev. 2014 May 10;9:10. doi: 10.1186/1749-8104-9-10 (PMC4077836; doi:10.1186/1749-8104-9-10)

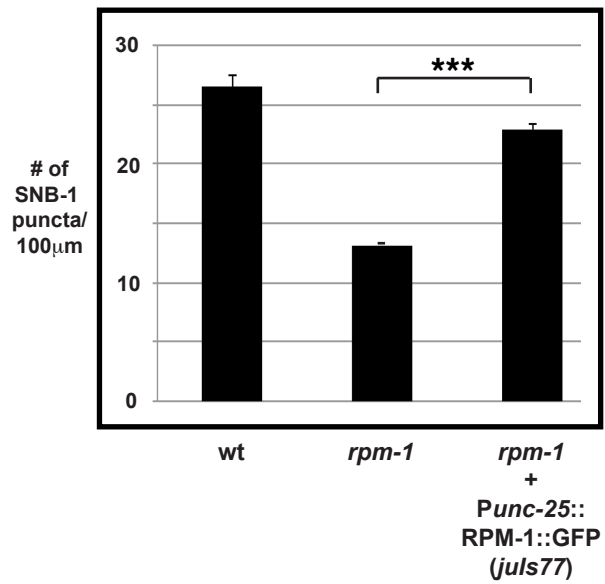

Supplement: Additional file 1 — juIs77 (P unc-25 RPM-1::GFP) rescues synapse formation defects in rpm-1 mutants.juIs1 (P unc-25 SNB-1::GFP) was used to visualize presynaptic terminals in the dorsal cords of animals with the indicated genotypes. SNB-1::GFP puncta were quantitated by scoring the number of puncta that were present per 100 μm of dorsal cord. Note that juIs77 (P unc-25 RPM-1::GFP rescues synapse formation defects caused by rpm-1 (lf). [file 1749-8104-9-10-S1.pdf]
